# Typical CIDP, distal variant CIDP, and anti-MAG antibody neuropathy: An ultra-high frequency ultrasound comparison of nerve structure

**DOI:** 10.1038/s41598-024-54452-8

**Published:** 2024-02-26

**Authors:** Angela Puma, Nicolae Grecu, Raluca Ș. Badea, Adeline Morisot, Roxana Zugravu, Mihai B. Ioncea, Michele Cavalli, Oana Lăcătuș, Andra Ezaru, Chorfa Hacina, Luisa Villa, Charles Raffaelli, Nicolas Azulay, Sabrina Sacconi

**Affiliations:** 1 Peripheral Nervous System and Muscle Department, Université Côte d’Azur, CHU Nice, 30 Voie Romaine, 06000 Nice, France; 2 Faculty of Medicine, UMR7370 CNRS, LP2M, Labex ICST, Université Nice Côte d’Azur, Nice, France; 3 Neurology Department, University Emergency Hospital Bucharest, 169 Splaiul Independentei, 050098 Bucharest, Romania; 4 Carol Davila University of Medicine and Pharmacy, 8 Bulevardul Eroii Sanitari, 050474 Bucharest, Romania; 5 Department of Public Health, University Hospital of Nice, Nice, France; 6 Service Médicine Polyvalente, Pôle Spécialités Médicales, CH Grasse, 28 Chemin de Clavary, 06180 Grasse, France; 7 Ultrasound Department, Université Côte d’Azur, CHU Nice, 30 Voie Romaine, 06000 Nice, France

**Keywords:** Biomarkers, Diseases, Medical research, Neurology, Signs and symptoms

## Abstract

To date, little is known about the usefulness of ultra-high frequency ultrasound (UHF-US, 50–70 MHz) in clinical practice for the diagnosis of dysimmune neuropathies. We present a prospective study aimed at comparing UHF-US alterations of nerves and fascicles in chronic inflammatory demyelinating polyradiculoneuropathy (CIDP), distal CIDP (d-CIDP) and anti-MAG neuropathy and their relationships with clinical and electrodiagnostic (EDX) features. 28 patients were included (twelve CIDP, 6 d-CIDP and 10 anti-MAG) and ten healthy controls. Each patient underwent neurological examination, EDX and UHF-US study of median and ulnar nerves bilaterally. UHF-US was reliable in differentiating immune neuropathies from controls when using mean and/or segmental nerve and/or fascicle cross-sectional area (CSA); furthermore, fascicle ratio (fascicle/nerve CSA) was a reliable factor for differentiating d-CIDP from other types of polyneuropathies. The fascicle CSA appears to be more increased in CIDP and its variant than in anti-MAG neuropathy. UHF-US offers information beyond simple nerve CSA and allows for a better characterization of the different forms of dysimmune neuropathies.

Chronic inflammatory demyelinating polyneuropathy (CIDP) is an immune-mediated polyneuropathy with a progressive course over at least 2 months. The diagnosis of CIDP is based on clinical signs and symptoms, demyelination electrophysiologic (EDX) criteria and paraclinical tests. Recently, the EAN/PNS have revised the classification of CIDP into “typical CIDP” and its variants (distal, multifocal, focal, motor and sensory CIDP)^1^. Distal CIDP (d-CIDP) is considered a variant of CIDP only when it is not associated with the presence of IgM dysglobulinemia with anti-myelin-associated glycoprotein (anti-MAG) activity^2^. Nerve ultrasound (US) changes in immune-mediated neuropathies have become one of the supporting criteria for the diagnosis of CIDP^1^. The most common finding is the enlargement of cross-sectional area (CSA) of nerves and roots^3^. Concerning anti-MAG neuropathy, US features have not been well defined, with some authors describing a swelling of the nerve in its most distal portion^4^, while others reporting more widespread changes^5^ or enlargement at entrapment sites.^6^ Although nerve size is the main parameter described in most studies, more attention is increasingly paid to the internal structure of the nerve. Some data are available for fascicle CSA (f-CSA) in CIDP^7^ but, to our knowledge, little is known about changes occurring at the level of the fascicle in d-CIDP and anti-MAG neuropathy. Our previous work has shown that Ultra High Frequency-US (UHF-US, 50 MHz) allows for better evaluation of the size and number of fascicles than classical probes, providing more detailed information on alterations of the internal nerve structure in patients with CIDP^8^. Based on this preliminary knowledge, we used an ultra-high frequency probe in order to distinguish between different dysimmune neuropathies. To this end, we analysed the nerve and fascicle CSA in patients with CIDP, d-CIDP and anti-MAG neuropathy.

## Methods

### Patients

Twenty-eight consecutive patients (20 men, 8 women) followed at the Neuromuscular Disease Center of Nice University for immune-mediated neuropathies and 10 healthy controls (3 men, 7 women) were enrolled between May 2018 and September 2019. The study was registered in the French clinical trial registry (IDRCB: 2016-A01603-48) and approved by the local ethics committee (Comité de Protection des Personnes Sud Méditerranée V). All methods were performed in accordance with the relevant guidelines and regulations. Written informed consent for participation was obtained from all patients. Patients with typical CIDP and d-CIDP had clinical features and EDX changes complying with the criteria proposed by the EFNS/PNS from 2010^9^. All patients with anti-MAG neuropathy had a monoclonal IgM gammopathy (monoclonal gammopathy of clinical significance, Waldenström's macroglobulinemia) with a peak of monoclonal proteins in the serum < 11 gr/dl. Anti-MAG antibody titers were assessed by an enzyme-linked immunosorbent assay (ELISA) and serum antibody activity was expressed as Bühlmann Titer Unit. All patients were treatment-naive. All patients underwent neurological evaluation and clinical severity was quantified with the Inflammatory Neuropathy Cause and Treatment Group (INCAT) disability scale, the Overall Neuropathy Limitations Scale (ONLS), and the Medical Research Council (MRC) sum score. Clinical evaluation, including INCAT, ONLS and MRC scores, were performed by the treating neurologists.

### Electrodiagnostic studies

EDX evaluation (Dantec Keypoint, Natus Medical Incorporated, Orlando, USA) was performed on all subjects between 1 and 2 months prior to the US. Four motor (tibial, peroneal, median, ulnar) and four sensory (superficial peroneal, sural, median, ulnar) nerves were examined bilaterally. The normative reference values used in our laboratory are summarized in Table 1. For each patient we ensured that skin temperature was at least 32 °C. An EDX score was created taking into consideration several criteria: for distal motor latency (DML) prolongation a score of 1 was assigned for a prolongation of 30–50%, 2 for a prolongation of 50–70%, and 3 for ≥ 70% of the upper limit of normal (ULN); for motor conduction velocity (MCV) reduction a score of 1 was given for a reduction of = 30%, 2 for a reduction between 30 and 50%, and 3 for a reduction of > 50% of the lower limit of normal (LLN); for compound motor action potential (CMAP) amplitude decrease a score of 1 was given for a reduction of ≤ 50%, a score of 2 for a reduction between 50 and 70%, a score of 3 for a reduction of ≥ 70%, and a score of 4 if CMAP was unobtainable; for conduction block (defined as ≥ 50% reduction of proximal relative to distal negative peak CMAP amplitude, excluding the tibial nerve and Erb’s point stimulation) a score of 1 was assigned; if EDX studies were considered normal a score of 0 was assigned (Table 1).

**Table 1:** EDX score.

	Score 1	Score 2	Score 3	Score 4	Normal values*
DML (ms)	30–50	50–70	≥ 70%	NA	MN ≤ 4
					UN ≤ 3,5
					TN et PN ≤ 5,5
MCV (m/s)	30	30–50	≥ 50%	NA	UL > 45
					LL > 40
cMAP (mV)	≤ 50	50–70	≥ 70%	NR	MN > 6
					UN > 7
					TN > 5
					PN > 3
CB	≥ 50%	NA	NA	NA	NA

*MN* median nerve, *UN* ulnar nerve, *TN* tibial nerve, *PN* peroneal nerve, *UL* upper limbs, *LL* lower limbs, *DML* distal motor latency, *MCV* motor conduction velocity, *cMAP* compound motor action potential, *CB* conduction block, *ms* milliseconds, *m/s* meter/second, *mV* millivolts, *NA* not applicable, *NR* no response

*values used in our laboratory.

### Ultrasound assessment

**Figure 1:**
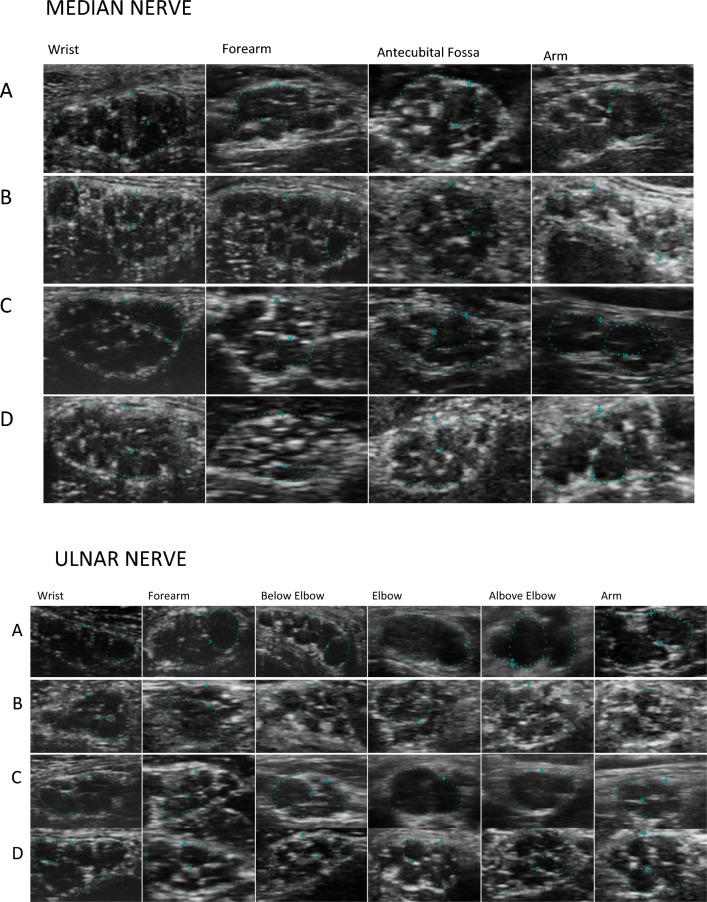
Median and Ulnar nerve in CIDP (**A**), in anti-MAG neuropathy (**B**), d-CIDP (**C**) and in healthy subject (**D**). In CIDP (**A**) thinning of perifascicular epineurium due to compression/dislocation from swollen fascicles. Reduced number of fascicles and swollen fascicles compared to healthy controls especially in more proximal nerve sites due to endoneuronal oedema. Focal/diffuse hypoechogenicity. In Anti-MAG neuropathy (B), reduced number of swollen fascicles with normal or slightly reduced perifascicular epineurium. Same or slightly reduced number of fascicles compared to healthy controls. Normal or slightly reduced echogenicity. In d-CIDP (**C**), normal number of swollen fascicles with normal or slightly reduced perifascicular epineurium. Normal or focal/diffuse hypoechogenicity.

Ultrasound evaluations of the peripheral nerves were performed with two very high-resolution probes (UHF48 and UHF70 MHz, Vevo, VD, Visualtronics, Toronto, Canada). The highest frequency probe, UHF70, was used with the “General” preset, with a 29–71 MHz frequency band. When using UHF70 exploration depth was limited to 7.5 mm. The second probe, UHF48, was used with the “General” preset, with a 20–48 MHz frequency band. When using UHF48 exploration depth was limited to 14.5 mm. We used the UHF70 whenever the depth of the explored nerve was below 7.5 mm, usually at the wrist and elbow, The median nerve (MN) and ulnar nerve (UN) were examined from the wrist to the middle-arm. The probe was adjusted to be perpendicular to the nerves, no pressure was applied, and a neutral position was adopted for each limb except for the examination of the ulnar nerve at the elbow, which was performed with the elbow flexed at 90°. Ultrasound examination of the nerve was performed bilaterally. The CSA for the MN was measured at the wrist, 10 cm from the distal wrist crease, at the antecubital fossa and mid-arm (Fig. 1). The CSA for the UN was measured at the wrist, 10 cm from the pisiform bone, at the elbow, 5 cm below and above the elbow and at mid-arm (Fig. 1). Assessment of nerve CSA (n-CSA) was performed on cross-sectional images using the manual tracking method by placing the cursor within the hyperechoic edge of the nerves. The depth function was not standardized but adjusted individually depending on the anatomy of the nerve at the examination site and the characteristics of the patient (amount of subcutaneous tissue) to allow visualization of the nerve in its entirety on cross-sections. The zoom function was not used. Fascicle identification and f-CSA measurement were performed on the same cross-sectional images used for n-CSA measurement. Fascicles were defined as hypoechoic areas surrounded by hyperechoic borders within the nerve and the CSA measurement was performed with the cursor placed within this hyperechoic margin^8^.

For each nerve, the largest f-CSA was measured and considered for statistical analysis. CSA reference values were obtained from the 10 healthy controls. Nerve and fascicle CSA were considered to be abnormal if 2 standard deviations (SD) greater than the upper limit. The fascicular ratio (FR) was calculated as f-CSA/n-CSA^10^.

To accommodate the potential variability in CSA distributions across different nerve segments and to enhance the diagnostic sensitivity of UHF-US in differentiating between dysimmune neuropathies, we also analyzed the largest nerve and fascicles CSA of the ulnar and median nerve for each pathology.

### Statistical analysis

Statistical analysis was performed using IBM SPSS Statistics for Windows, Version 26.0 (Armonk, NY: IBM Corp). Continuous variables with normal distribution are presented as mean ± standard deviation, while those with non-normal distribution are presented as median (interquartile range). Categorical variables are reported as the rate (percentage). For determining if there were differences between the four groups (controls, CIDP, d-CIDP, anti-MAG) concerning continuous data, one-way ANOVA tests and Kruskal–Wallis One-Way ANOVA on Ranks were conducted. Outliers were kept in the analysis, as they depicted true clinical scenarios. For determining the normality of data for each of the studied group, the Shapiro–Wilk test was performed. The homogeneity of variances was assessed by Levene’s test of homogeneity of variances. For data for which the homogeneity of variances was violated, Welch ANOVA was used; in this case, post-hoc analysis was interpreted using the results of the Games-Howell post-hoc test. In cases in which the homogeneity of variances was met, post-hoc analysis was interpreted using the Tukey–Kramer post hoc test. When Kruskal–Wallis test was performed, post-hoc analysis was made using Dunn’s (1964) procedure with a Bonferroni correction for multiple comparisons. For assessing the relationships between variables, Pearson correlation test was performed. In case of non-linearity, Spearman’s rank-order correlation test was run.

## Results

Thirty-eight participants were included in the current study: 12 with typical CIDP, 6 with distal variant CIDP (d-CIDP), and 10 with demyelinating neuropathy associated with anti-MAG antibodies (anti-MAG). Ten healthy controls were also included. Fifteen of those participating in the study (39.5%) were female, and 23 (60.5%) were male; there were more females in the control group than in the study group (70% vs. 28.6%).

Mean age of patients was 62.79 years (± 12.83), the youngest group consisting of d-CIDP patients (mean age 50 ± 12.72 years), followed by CIDP (mean age 62.3 ± 12.4 years), and anti-MAG patients (mean age 68 ± 7.51 years) (Table 1).

The duration between the onset of neurological symptoms and first medical evaluation differed significantly between the three polyneuropathy groups, patients with anti-MAG antibodies having the longest duration of symptoms (19 months), followed by patients with CIDP (6.5 months), and by those with d-CIDP (3 months), χ2(2) = 8.942, *p* = 0.011. Post-hoc analysis revealed statistically significant differences in symptoms duration between the patients with d-CIDP and the ones with anti-MAG antibodies (*p* = 0.008) The demographic characteristics, clinical and electrophysiological scores are summarized in Table 2.

**Table 2:** Clinical, demographical, and electrodiagnostic data.

	Control	Anti-MAG	CIDP	d-CIDP	*p*
Age (± SD)	54.2 ± 19.37	68.2 ± 7.11	62.33 ± 13.48	54.67 ± 16.13	0.138
	(30–82)	(55–80)	(31–77)	(33–79)	
Gender					
F	7 (70%)	2 (20%)	4 (33.3%)	2 (33.3%)	0.153
M	3 (30%)	8 (80%)	8 (66.7%)	4 (66.7%)	
Anti-MAG antibodies					
> 50,000 BTU	NA	6 (60%)	NA	NA	
< 50,000 BTU	NA	4 (40%)	NA	NA	
Duration of symptoms* (Months, IQR)	NA	19.5 (7–36)	6.5 (5–24.5)	3 (3–5)	.011*
INCAT (± SD)	NA	3.11 ± 2.26	3.92 ± 3.42	4.67 ± 4.63	0.116
ONLS (± SD)	NA	2.56 ± 2.00	3.08 ± 2.57	3.67 ± 4.08	0.441
MRC (IQR)	NA	60, (57.9–60)	60, (54.9–60)	55.8, (44.2–60)	0.263
EDX (± SD)	NA	21.00 ± 4.77*	16.00 ± 12.91	7.33 ± 3.72*	0.035
Height (± SD)	168.70 ± 10.68	171.40 ± 8.19	170.83 ± 9.80	181.40 ± 9.60	0.126
Weight (± SD)	63.60 ± 9.69	75.40 ± 11.39	72.00 ± 18.16	77.83 ± 18.22	0.212

*Statistically significant difference

*M* male, *F* female, *INCAT* Inflammatory Neuropathy Cause and Treatment Group (INCAT) disability scale, *IQR* interquartile range, *NA* not applicable, *ONLS* Overall Neuropathy Limitations Scale, *SD* Standard deviation.

### Clinical and electrophysiological tests

Patients with d-CIDP had the highest INCAT (4.67 ± 4.63) and ONLS (3.67 ± 4.08) scores, but also the lowest EDX score (7.33 ± 3.72). They were followed by CIDP patients (average INCAT, ONLS, and EDX scores of 3.92 ± 3.42, 3.08 ± 2.57, and 16 ± 12.91, respectively), and by anti-MAG patients (mean INCAT, ONLS, and EDX scores of 3.11 ± 2.26, 2.56 ± 2, and 21 ± 4.77, respectively). MRC sum score was highest in CIDP and anti-MAG patients. However, the only statistically significant difference between the three groups was for the EDX score, with post-hoc analysis revealing that anti-MAG patients had significantly higher EDX scores than d-CIDP patients (increase of EDX score with 13.66, (95% CI, 7.83–19.5)). The demographic characteristics, clinical and electrophysiological scores are summarized in Table 2.

A Spearman's rank-order correlation was run to assess the relationship between EDX score and mean CSA of nerves and fascicles of ulnar and median nerves. There was a statistically significant, strong positive correlation between EDX scores and overall MN n-CSA r_s_(25) = 0.457, *p* = 0.017, and between EDX score and mean UN n-CSA r_s_(25) = 0.568, *p* = 0.002. There was no correlation between EDX score and f-CSAs.

When looking at the relationship between the duration of the disease and the mean CSA of nerves and fascicles of ulnar and median nerves, there was a statistically significant correlation between the duration of the disease and the median nerve CSA at the mid-arm, patients with older disease having a larger CSA of the median nerve, r_s_ (28) = 0.379, *p* = 0.047.

### Ultrasound assessment of median and ulnar nerves and fascicles CSA

**Figure 2:**
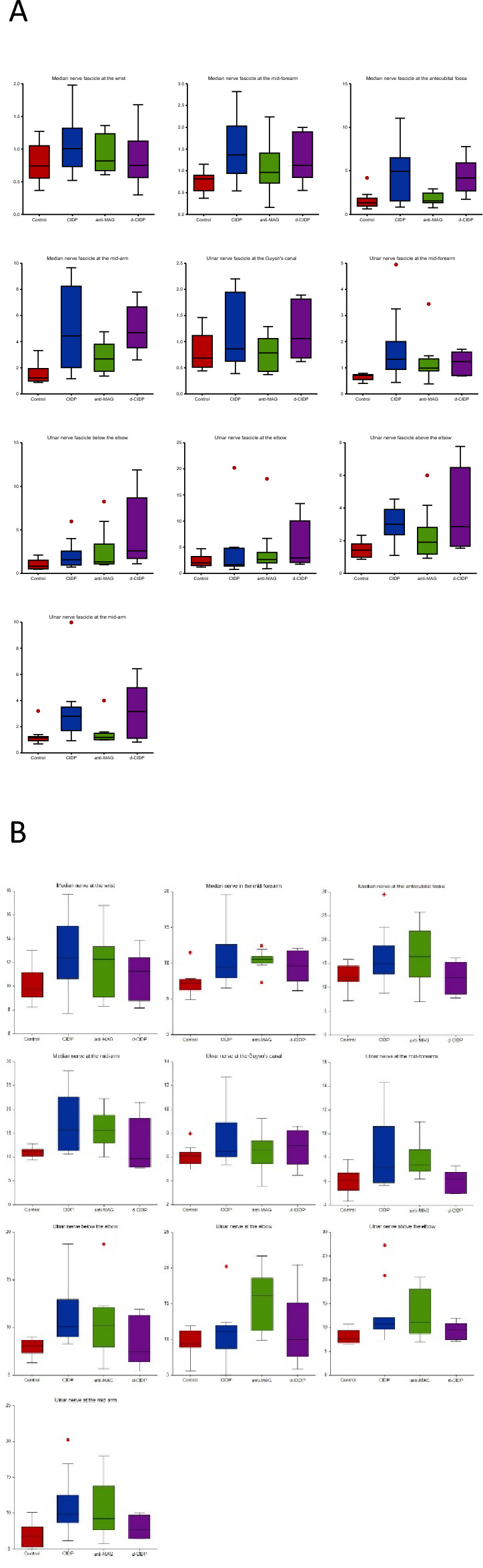
Mean nerve CSA values (**A**: right; **B**: left) for the median and ulnar nerve in controls and neuropathy patients.

**Figure 3:**
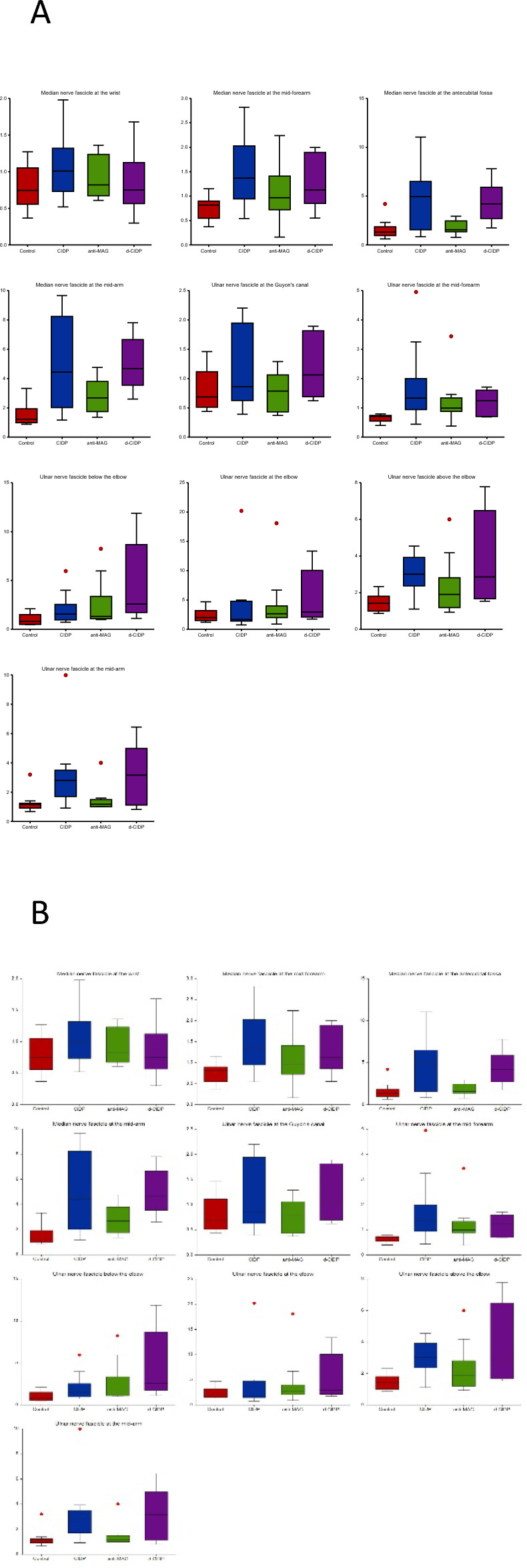
Mean fascicle CSA values (**A**: right; **B**: left) for the median and ulnar nerve in controls and neuropathy patients.

Mean MN and UN n-CSA and f-CSA values are represented in Figs. 2 and 3. Detailed results can be found in Table 3.

**Table 3:** Differences between nerve and fascicle cross-sectional area (in mm^2^) for median and ulnar nerve in controls and patients with immune neuropathies.

	Control	anti-MAG	CIDP	d-CIDP	*p*
MN n-CSA wrist	10.14 ± 1.42	11.77 ± 2.67	12.80 ± 3.00	10.92 ± 2.07	0.093
MN n-CSA mid-forearm*	7.25, (IQR 6.2–7.7)	10.51, (IQR 9.9–11.2)	9.03, (IQR 7.6–13.6)	11 (IQR 7–11.8)	**0.009**
MN n-CSA antecubital fossa	12.50 ± 2.55	16.87 ± 6.14	16.29 ± 5.49	11.99 ± 3.70	0.084
MN n-CSA mid-arm*	11.05 ± 0.99	15.94 ± 3.93	17.30 ± 6.10	12.26 ± 5.70	**0.003**
MN f-CSA wrist	0.78 ± 0.30	0.92 ± 0.29	1.04 ± 0.41	0.84 ± 0.46	0.339
MN f-CSA mid-forearm*	0.76 ± 0.22	1.06 ± 0.57	1.47 ± 0.69	1.27 ± 0.56	**0.021**
MN f-CSA antecubital fossa*	1.59 ± 1.04	1.79 ± 0.71	4.84 ± 3.16	4.37 ± 2.07	**0.007**
MN f-CSA mid-arm*	1.53 ± 0.78	2.80 ± 1.15	5.04 ± 3.09	4.98 ± 1.82	** < 0.003**
UN n-CSA Guyon	6.10, (IQR 5.4–6.4)	6.61, (IQR 5.4–6.9)	6.48, (IQR 5.8–9.7)	6.95, (IQR 5.1–7.6)	0.347
UN n-CSA mid-forearm*	6.04 ± 1.08	7.83 ± 1.48	8.53 ± 2.94	5.94 ± 0.98	**0.012**
UN n-CSA below elbow*	7.89 ± 0.89	10.63 ± 3.60	11.10 ± 3.01	8.56 ± 2.64	**0.040**
UN n-CSA elbow*	9.49 ± 1.76	15.33 ± 4.26	11.00 ± 3.73	11.27 ± 5.11	**0.010**
UN n-CSA above elbow*	7.72, (IQR 6.7–9.3)	10.93, (IQR 8.4–18.4)	10.73, (IQR 9.4–14.3)	8.76, (IQR 7.5–10.8)	**0.014**
UN n-CSA mid-arm*	6.92 ± 1.76	10.42 ± 4.24	11.17 ± 4.10	8.00 ± 1.64	**0.031**
UN f-CSA Guyon	0.68, (IQR 0.5–1.1)	0.72, (IQR 0.4–1.1)	0.86, (IQR 0.7–2)	1.25 (IQR 0.71.8)	0.245
UN f-CSA mid-forearm*	0.71, (IQR 0.5–0.7)	0.99, (IQR 0.8–1.3)	1.33, (IQR 0.9–2.3)	1.09, (IQR 0.7–1.6)	**0.004**
UN f-CSA below elbow*	0.82, (IQR 0.5–1.5)	1.32, (IQR 1–2.4)	1.86 (IQR 0.9–2.4)	3.87, (IQR 1.7–8.6)	**0.019**
UN f-CSA elbow	1.98, (IQR 1.5–3.2)	3, (IQR 2–3)	1.66, (IQR 1.4–4.7)	5.56, (IQR 2–11)	0.422
UN f-CSA above elbow*	1.42, (IQR 1–1.8)	1.82, (IQR 1.8–2.3)	3, (IQR 2.1–4)	3.47, (IQR 1.7–6.5)	0.**010**
UN f-CSA mid-arm*	1.16, (IQR 0.9–1.2)	1.21, (IQR 1–1.4)	2.79, (IQR 1.7–3.4)	3.3, (IQR 1.1–5)	0.**010**
Average MN CSA*	10.23 ± 1.10	13.74 ± 2.30	14.19 ± 3.85	11.18 ± 2.69	**0.002**
Average MN f-CSA*	1.17 ± .41	1.65 ± .39	3.19 ± 1.59	2.86 ± .94	** < 0.001**
Average UN CSA*	7.51, (IQR 6.58.2)	10.81, (IQR 7.9–13.8)	9.29, (IQR 8.32–10.8)	8.24, (IQR 7.3–9.6)	**0.005**
Average UN f-CSA*	1.25, (IQR 1–1.4)	1.58, (IQR 1.4–2.5)	1.94, (IQR 1.5–2.9)	2.59, (IQR 2–4.6)	**0.001**
MN FR wrist	0.07 ± 0.02	0.07 ± 0.02	0.08 ± 0.02	0.07 ± 0.04	0.964
MN FR mid-forearm	0.10 ± 0.03	0.10 ± 0.05	0.12 ± 0.05	0.13 ± 0.07	0.509
MN FR antecubital fossa*	0.12 ± 0.06	0.12 ± 0.05	0.29 ± 0.19	0.41 ± 0.31	**0.021**
MN FR mid-arm*	0.10, (IQR 0.1–0.2)	0.14, (IQR 0.1–0.3)	0.30, (IQR 0.1–0.4)	0.57, (IQR 0.3–0.9)	**0.006**
UN FR Guyon	0.11, (IQR 0.08–0.2)	0.12, (IQR 0.06–0.2)	0.13, (IQR 0.1–0.2)	0.19, (IQR 0.1–0.2)	0.506
UN FR mid-forearm*	0.12, (IQR 0.07–0.1)	0.13, (IQR 0.1–0.2)	0.16, (IQR 0.1–0.2)	0.18, (IQR 0.1–0.2)	**0.041**
UN FR below elbow*	0.11, (IQR 0.07–0.2)	0.18, (IQR 0.1–0.3)	0.16, (IQR 0.09–0.2)	0.65, (IQR 0.2–1)	**0.049**
UN FR elbow	0.21, (IQR 0.2–0.3)	0.20, (IQR 0.1–0.3)	0.18, (IQR 0.1–0.4)	0.40, (IQR 0.2–0.7)	0.186
UN FR above elbow	0.18 ± 0.07	0.17 ± 0.08	0.26 ± 0.12	0.42 ± 0.33	0.171
UN FR mid-arm*	0.18 ± 0.06	0.13 ± 0.06	0.27 ± 0.10	0.40 ± 0.34	**0.024**
Largest MN n-CSA*	13.08 (1.75)	18.51 (5.59)	18.53 (5.59)	14.42 (4.91)	**0.018**
Largest MN f-CSA*	1.89 (1.10)	3.10 (0.88)	6.08 (3.21)	5.14 (1.66)	** < 0.001**
Largest UN n-CSA*	9.80, IQR 8.99–11.19	17.57, IQR 11.19–20.49	11.97, IQR 11.12–13.86	11.04, IQR 9.31–15.11	**0.006**
Largest UN f-CSA*	2.34, IQR 1.563.36	3.00, IQR 2.35–6.56	3.65, IQR 2.91–4.90	5.81, 3.01–12.25	**0.037**

Significance values in Bold.

*Asterisks show statistical significance.

Statistically significant differences between the four studied groups were found for: the average MN n-CSA, F (3, 34) = 4.884, *p* = 0.006, and MN f-CSA, F (3, 34) = 8.950, *p* =  < 0.001; the MN n-CSA at the mid-arm level F (3, 34) = 4.154, *p* = 0.013, ω2 = 0.199, and at the mid-forearm χ2(3) = 11.485, *p* = 0.009; the MN f-CSA in the mid-forearm F (3, 33) = 3.175, *p* = 0.037, ω2 = 0.150, the antecubital fossa F (3, 34) = 6.671, *p* = 0.001, ω2 = 0.309, and in the mid-arm F (3, 34) = 6.880, *p* =  < 0.001, ω2 = 0.317.

For the ulnar nerve, there were significant differences in the n-CSA between the four groups at the mid-forearm site F (3, 32) = 3.944, *p* = 0.017, ω2 = 0.197, below elbow F (3, 33) = 3.106, *p* = 0.040, ω2 = 0.146, at the elbow F (3, 33) = 4.451, *p* = 0.010, ω2 = 0.219, above elbow χ2(3) = 10.669, *p* = 0.014, and in the mid-arm site F (3, 31) = 3.363, *p* = 0.031, ω2 = 0.168, as well in the f-CSA when measured in the mid-forearm site χ2(3) = 13.523, *p* = 0.004, below the elbow χ2(3) = 9.926, *p* = 0.019, above the elbow χ2(3) = 101.300, *p* = 0.010, and in the mid-arm χ2(3) = 11.341, *p* = 0.010. Significant differences between the four groups were also found when analyzing the average UN n-CSA, χ2(3) = 12.686, *p* = 0.005, and the average UN f-CSA χ2(3) = 16.104, *p* = 0.001.

When looking at the largest MN and UN CSA as measured by the UHF-US, significant between-group differences were seen for the studied nerves, suggesting substantial variations in nerve and fascicle enlargements in CIDP, d-CIDP and anti-MAG neuropathy compared to controls. Eta-squared values indicated a strong effect size for f-CSA (η^2^ = 0.435), pointing to its potential as a sensitive biomarker for distinguishing neuropathic changes. (Table 3).

### CIDP vs. controls

Post hoc analysis showed that CIDP patients had higher MN n-CSA, and statistical significance was found for the n-CSA at the mid arm (increase of 6.25 mm^3^ (95% CI, 0.91–11.58), *p* = 0.021), as well as for the MN f-CSA at the mid-forearm (increase of 0.71 mm^3^, 95% CI, 0.06–1.37, *p* = 0.031), antecubital fossa (increase of 3.24 mm^3^, 95% CI, 0.42–6.07, *p* = 0.022), and mid-arm levels (increase of 3.50 mm^3^, 95% CI, 0.77–6.24, *p* = 0.011). Overall mean MN n-CSA and mean MN f-CSA were significantly higher in CIDP patients, with an increase of 3.95 mm^3^, (95% CI, 0.53–7.37), *p* = 0.022, and respectively 2.02 mm^3^, (95% CI, 0.83–3.20), *p* < 0.001.

CIDP patients also had larger UN n-CSA, with a significant increase of the CSA of: 3.01 mm^3^, *p* = 0.033 above the elbow; 3.2 mm^3^ (95% CI, 0.005–6.40), *p* = 0.049 below the elbow; and 4.24 mm^3^ (95% CI, 0.24–8.24), *p* = 0.034 at the mid-arm level. UN f-CSA was also significantly larger in CIDP patients than in controls, with an increase of 0.62 mm^3^ in the mid-forearm, *p* = 0.002, and of 1.16 mm^3^ in the mid-arm site, p = 0.014. Overall mean UN n-CSA and mean UN f-CSA were significantly higher in CIDP patients than controls, with an increase of 1.78 mm^3^, *p* = 0.013, and respectively 0.69 mm^3^, *p* = 0.002.

These differences were consistent upon analysis of the largest median nerve (MN) n-CSA and f-CSA, with patients diagnosed with CIDP exhibiting a notably larger MN n-CSA (increase of 5.44 mm^3^, 95% CI (0.05, 10.84), *p* = 0.047) and f-CSA (increase of 4.19 mm^3^, 95% CI (1.71, 6.67), *p* < 0.001) compared to control subjects. Similar patterns were observed for the ulnar nerve (UN) n-CSA, where patients with CIDP demonstrated a significant increase of 2.17 mm^3^, *p* = 0.032 relative to controls. However, while the largest UN f-CSA was higher in the CIDP group compared to controls, this difference did not reach statistical significance (*p* = 0.182).

### Anti-MAG vs. controls

Anti-MAG patients had significantly larger MN n-CSA in the mid-forearm (increase of 3.26 mm^3^, *p* = 0.007) and in the mid-arm (increase of 4.89 mm^3^ (95% CI, 0.972–8.81), *p* = 0.015), while MN f-CSA was significantly larger only in the mid-arm site (increase of 1.27 mm^3^ (95% CI, 0.005–2.54), *p* = 0.049). Overall average MN n-CSA was significantly higher in patients with anti-MAG than controls (increase of 3.51 mm^2^, *p* = 0.004).

Anti-MAG patients also had larger UN n-CSA in the mid-forearm (increase of 1.79 mm^3^, (95% CI, 0.142–3.45), *p* = 0.031), at the elbow (increase of 5.84 mm^3^ (95% CI, 1.31–10.36), *p* = 0.007), and above the elbow (increase of 3.21 mm^3^). Overall mean UN n-CSA and mean UN f-CSA were significantly higher in patients with anti-MAG than controls, with an increase of 3.3 mm^2^, *p* = 0.002, and respectively 0.33 mm^2^, *p* = 0.044.

When focusing on the largest CSA measurements of the studied nerves, it was observed that patients with anti-MAG neuropathy exhibited a trend toward larger MN n-CSA and f-CSA than controls, with an increase in MN n-CSA of 5.43 mm^3^; however, this difference did not reach statistical significance (95% CI (−0.20, 11.06), *p* = 0.064). Similarly, the increase in MN f-CSA of 1.21 mm^3^ (95% CI (−1.37, 3.81), *p* = 1.00) and the UN f-CSA of 0.66 mm^3^ (*p* = 0.159) were larger in the anti-MAG group compared to controls but were not statistically significant. In contrast, the largest ulnar nerve (UN) n-CSA measurements proved to be a more robust differentiator, with patients with anti-MAG neuropathy displaying a statistically significant larger UN n-CSA (increase of 7.77 mm^3^, *p* = 0.001) compared to the control group.

### D-CIDP vs. control

While no statistically significant differences were found for the MN and UN n-CSA, d-CIDP patients had significantly larger MN f-CSAs at the mid-arm site (increase of 3.44 mm^3^ (95% CI, 0.739–6.15), *p* = 0.017) and UN f-CSAs below the elbow (increase of 3.05 mm^3^, *p* = 0.021). Furthermore, mean f-CSAs were significantly higher in d-CIDP patients, with an increase of 1.69 mm^3^, (95% CI, 0.26 to 3.12), *p* = 0.015 for the MN, and of 1.34 mm^3^, *p* < 0.001 for the UN respectively. Highest values of the CSAs of the studied nerves were also increased in patients with d-CIDP when compared with controls. Nonetheless, statistical significance was reached only in the case of the MN f-CSA (increase of 3.25 mm^3^, 95% CI (0.25, 6.25), *p* = 0.027) and UN f-CSA (increase of 3.47 mm^3^, *p* = 0.007) in d-CIDP patients when compared with controls.

### Differences between neuropathies

When testing for differences between the three neuropathy groups, CIDP patients had significantly larger f-CSAs compared to anti-MAG patients: for the MN overall (increase of 1.54 mm^3^ (95% CI, 0.13–2.95), *p* = 0.031), and in the antecubital fossa (increase of 3.04 mm^3^ (95% CI, 0.54–5.54), *p* = 0.017), and for the UN when measured in the midarm site (increase of 1.58 mm^3^, *p* = 0.017). Anti-MAG patients had significantly larger UN n-CSA than d-CIDP patients when measured in the mid-forearm site, with an increase of the n-CSA of 1.89 mm^3^ (95% CI, 0.16–3.61), *p* = 0.032. Further, when focusing on the largest CSA measurements within the study, CIDP patients had a notably larger MN f-CSA (increase of 2.97 mm^3^, 95% CI (0.49, 5.46), *p* = 0.012) compared to those with anti-MAG neuropathy, indicating distinct patterns of nerve involvement between these conditions.

### Role of the FR in differentiating between groups

When comparing with controls, CIDP patients had higher MN FR at the antecubital fossa, with an increase of 0.172, (95% CI, 0.001–0.343), *p* = 0.047, d-CIDP patients had higher MN FR at the mid-arm site (increase of 0.47, *p* = 008), and UN FR below the elbow (increase of 0.54, *p* = 0.038).When testing for differences between the three neuropathy groups, CIDP patients had higher FR compared to anti-MAG patients: for the MN at the antecubital fossa (increase of 0.177, (95% CI, 0.02–0.32), *p* = 0.023) and for the UN at the mid-arm site (increase of 0.138, (95% CI, 0.38–0.23), *p* = 0.007).

## Discussion

In recent years, ultrasound assessment of peripheral nerves in chronic inflammatory neuropathies (CIN) is considered a valuable diagnostic tool^11^. Peripheral nerve ultrasound parameters are mainly limited to the quantitative assessment of nerve CSA^12,13^. Reference values have been proposed with high-frequency (12–20 MHz) ultrasound probes^14^. Our study proposes the first normative values of n-CSA and f-CSA using a UHF probe (Table 3). No substantial differences were found between the n-CSA values obtained with our probe and the more commonly used high-frequency probe^7,14^ . What is quite surprising, however, is the difference when calculating f-CSA with the two probes. Grimm et al. previously established f-CSA values in healthy persons and in patients with CIN using a HF probe^7^. These values are significantly higher than those found in our study, probably due to the difficulty in distinguishing the different fascicles with the conventional probe^15^. As mentioned in our previous work, the UHF probe allows for better visualization of the fascicles, better characterization of their size and morphology, as well as for an accurate estimate of their number.^8^. The number of fascicles increased in the median nerve from the axilla to the wrist, while it remained stable in the ulnar nerve with a reduction in number only at the elbow, in perfect accord with anatomical studies^16^.

**Figure 4:**
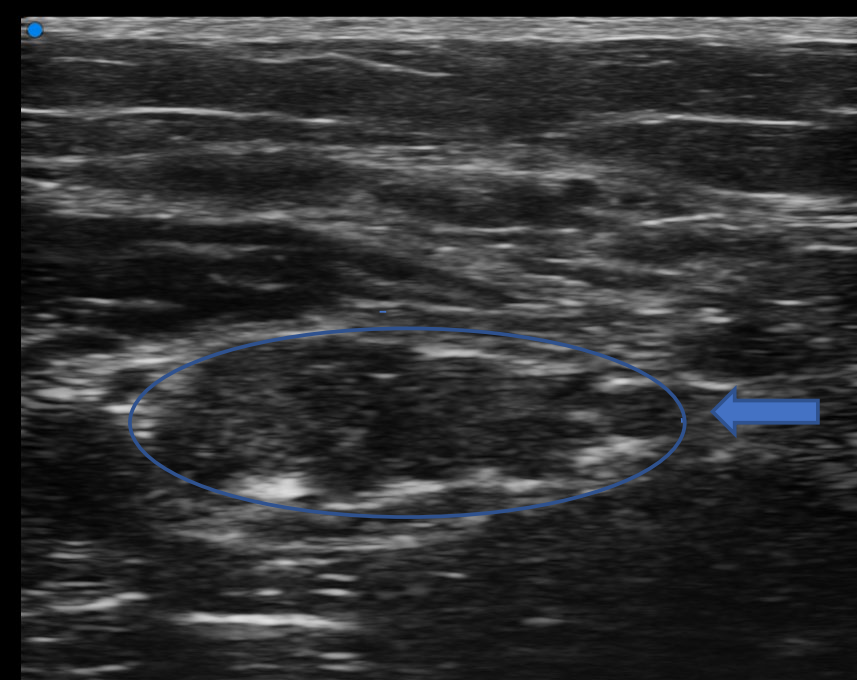
Median nerve in the antecubital fossa. The image shows fusion of neighboring fascicles with creation of a single fascicular plexus. Fascicles are swollen and hypoechogenic due to endoneural edema. Areas of focal hyperechogenicity due to fragmentation of the epineurium and endoneurium.

In recent years, a standardized ultrasound approach to assess the different components of the peripheral nerve, combined with quantitative assessment has been increasingly proposed^8,15,17^. Changes in the internal structure, which are consistent with loss of fascicular pattern due to intraneural edema (Fig. 4) and fibrosis, have been considered signs of nerve pathology on both US and MRI^10,18^. The most prominent US morphological changes in CIDP are multifocal nerve swellings and nonhomogeneous fascicular structure^19,20^. Our patients showed a similar pattern, with an enlargement of both the n-CSA and the f-CSA, more significantly in the proximal segments of the nerve (Fig. 1). In fact, if we consider all patients as a single dysimmune neuropathy group, a diffuse, but non-homogenous, increase in n-CSA was the uniting feature, more so in the proximal nerve segments. The analysis of the individual groups, on the other hand, revealed somewhat different information. For CIDP, both median and ulnar nerves showed an increase in n-CSA and f-CSA in the proximal segments, with f-CSAs also enlarged in the more distal segment, starting from mid-forearm. On the other hand, in MAG antibody neuropathy, nerve enlargement is also seen in distal segments, without f-CSA increase. Analysis of the larger fascicular CSA allows one to clearly distinguish CIDP from anti-MAG neuropathy. To have an overall picture of these changes, the FR would be a suitable parameter, and based on our data a FR greater than 0.30 would be in favor of CIDP or its distal variant. This would suggest a different involvement of the different nerve structures by the specific inflammatory and immune mechanisms, which relates to the well-known clinical, EDX and treatment response differences of three different types of neuropathies^17^. Recent data has shown that the blood nerve barrier (BNB) is also subject to contrasting changes in the three neuropathies. The inflammatory process in CIDP and in d-CIDP leads to disruption of the tight junctions (TJ)^21^, while penetration of anti-MAG IgM antibodies through the BNB occurs without tight junction disruption and without increased permeability to small molecules (possibly through transcytosis)^22^. Since the TJs of endoneurial vessels are preserved, this would possibly explain the more reduced fascicular swelling.

In d-CIDP, n-CSA values were overall lower than in the other neuropathies, while f-CSA were significantly increased, especially in proximal median nerve segments. Literature data regarding US in d-CIDP is scarce, due probably to two factors: firstly, most studies did not differentiate between subtypes or only included typical CIDP, and secondly a more definite definition was only recently made^1^. One report highlights diffuse n-CSA enlargement, especially in the distal sensory segments^23^. As far as our results are concerned, the fact that the n-CSA was not increased is not particularly surprising. This subgroup had a significantly shorter disease duration compared to the other groups and it has been shown that US changes are more evident the longer the patient's clinical history^20^. Also in our study, patients with a longer disease duration had a significantly larger fascicle area of the median nerve in the middle arm. On the other hand, these data seem to strengthen our hypothesis that in the case of CIDP and its distal variant, the inflammatory process electively affects the fascicles earlier in the disease and later the entire nerve.

In our study, no statistically significant association was found between clinical scores and the various groups of neuropathies. The absence of differences in clinical scores and different types of neuropathies was also noticed by Merola et al., while analyzing patients with CIDP and multifocal motor neuropathy^24^. However, an increase in mean MN et UN n-CSA was strongly correlated with an increase in EDX score, indicating that nerve enlargement is a marker of demyelination.^20,25^.

The main limitations of our study are the small number of subjects in each subgroup and the different disease duration in each case. Furthermore, there is a predominance of women in the control group compared to the CIDP and anti-MAG group—however, we believe this is not a true limitation per se, as a statistically significant gender-related difference in MN and UN CSA has never been demonstrated^26^.

In conclusion, we believe our findings show the potential of UHF-US in differentiating between immune neuropathies, with several markers of internal nerve structure being of particular interest. Changes in fascicle size without or with increased n-CSA have already been described in the CIN^27^. The use of UHF probes allows better visualization of the nerve's internal structure and, based on our data, we could hypothesize that an increase in f-CSA with or without an increase in n-CSA would point the diagnosis towards CIDP or a variant thereof, whereas an increase in n-CSA even in the most distal parts of the nerve without an increase in f-CSA could be a marker of anti-MAG neuropathy. Further studies are needed to consolidate our findings and establish UHF-US as a useful biomarker to distinguish between immune neuropathies.

## Data Availability

The datasets generated during and/or analyzed during the current study are available from the corresponding author on reasonable request.
